# Inducibility of Plant Secondary Metabolites in the Stem Predicts Genetic Variation in Resistance Against a Key Insect Herbivore in Maritime Pine

**DOI:** 10.3389/fpls.2018.01651

**Published:** 2018-11-21

**Authors:** Xosé López-Goldar, Caterina Villari, Pierluigi Bonello, Anna Karin Borg-Karlson, Delphine Grivet, Rafael Zas, Luís Sampedro

**Affiliations:** ^1^Misión Biológica de Galicia, Consejo Superior de Investigaciones Científicas, Pontevedra, Spain; ^2^Department of Plant Pathology, The Ohio State University, Columbus, OH, United States; ^3^Ecological Chemistry Group, Department of Chemistry, Royal Institute of Technology, Stockholm, Sweden; ^4^Department of Forest Ecology and Genetics, Forest Research Centre, INIA, Madrid, Spain; ^5^Daniel B. Warnell School of Forestry and Natural Resources, University of Georgia, Athens, GA, United States; ^6^Sustainable Forest Management Research Institute, INIA-University of Valladolid, Palencia, Spain

**Keywords:** genetic variation, herbivory, inducibility, maritime pine, plant secondary metabolites (PSM), phenolics, resistance, terpenes

## Abstract

Resistance to herbivores and pathogens is considered a key plant trait with strong adaptive value in trees, usually involving high concentrations of a diverse array of plant secondary metabolites (PSM). Intraspecific genetic variation and plasticity of PSM are widely known. However, their ecology and evolution are unclear, and even the implication of PSM as traits that provide direct effective resistance against herbivores is currently questioned. We used control and methyl jasmonate (MJ) induced clonal copies of genotypes within families from ten populations of the main distribution range of maritime pine to exhaustively characterize the constitutive and induced profile and concentration of PSM in the stem phloem, and to measure insect herbivory damage as a proxy of resistance. Then, we explored whether genetic variation in resistance to herbivory may be predicted by the constitutive concentration of PSM, and the role of its inducibility to predict the increase in resistance once the plant is induced. We found large and structured genetic variation among populations but not between families within populations in resistance to herbivory. The MJ-induction treatment strongly increased resistance to the weevil in the species, and the genetic variation in the inducibility of resistance was significantly structured among populations, with greater inducibility in the Atlantic populations. Genetic variation in resistance was largely explained by the multivariate concentration and profile of PSM at the genotypic level, rather than by bivariate correlations with individual PSM, after accounting for genetic relatedness among genotypes. While the constitutive concentration of the PSM blend did not show a clear pattern of resistance to herbivory, specific changes in the chemical profile and the increase in concentration of the PSM blend after MJ induction were related to increased resistance. To date, this is the first example of a comprehensive and rigorous approach in which inducibility of PSM in trees and its implication in resistance was analyzed excluding spurious associations due to genetic relatedness, often overlooked in intraspecific studies. Here we provide evidences that multivariate analyses of PSM, rather than bivariate correlations, provide more realistic information about the potentially causal relationships between PSM and resistance to herbivory in pine trees.

## Introduction

Resistance to herbivores and pathogens has been widely recognized as a key plant trait with strong adaptive value (Futuyma and Agrawal, 2009), particularly at early stages of development (Goodger et al., 2013). In long-lived plants with high apparency, like trees, resistance against pests and pathogens usually relies on high concentrations of a diverse array of plant secondary metabolites (PSM) (Feeny, 1976; Wiggins et al., 2016). Such chemical cocktail generally produces dose dependent effects, where higher concentrations in specific plant tissues targeted by herbivores results in reduced enemy performance and/or plant damage (Zhao et al., 2011b). These chemical defenses are expensive to produce and maintain (Gershenzon, 1994; Sampedro et al., 2011a). All these features are certainly present in conifers, whose higher concentrations of terpenes and phenolics are commonly associated with increased resistance against insect herbivores (Phillips and Croteau, 1999; Witzell and Martín, 2008; Schiebe et al., 2012).

Within conifer species, variation in defensive PSM is usually difficult to explain based exclusively on an arms race between trees and their biotic aggressors because many other factors often affect plant–organism interactions (Moore et al., 2013). For example, heterogeneity in the biotic and abiotic environment (Lombardero et al., 2000; Sampedro et al., 2010), the large, repetitive, and idiosyncratic structure of conifer genomes (Theis and Lerdau, 2003), trade-offs with other life functions (Sampedro et al., 2011a; Moreira et al., 2014) and other evolutionary forces, such as gene flow and genetic drift (Kremer et al., 2012), can all be involved in maintaining large intraspecific variation in PSM as well as in resistance. Phylogenetic analyses has been widely used in recent years (Rasmann and Agrawal, 2011; Pellissier et al., 2012; Moreira et al., 2014; Carrillo-Gavilán et al., 2015) to control for the statistical dependence among related species in macroecology (Freckleton et al., 2002), leading to the identification of imprinted historical events in contemporary observed traits (Pagel, 1999). At the intraspecific level, demographic processes, habitat fragmentation, gene flow and local adaptation can also greatly influence the variation in a species’ traits among and within populations (Savolainen et al., 2007; De-Lucas et al., 2009), making necessary to account for the genetic structure of populations and the relative kinship among genotypes within families when exploring trait-trait or trait-environment associations (Yu et al., 2006).

Evidence is accumulating that PSM are highly plastic, as most plant species respond with large changes in the concentration and profile of PSM to the perception of a biotic threat, such as a pathogen or a herbivore (Karban et al., 1999; Heil, 2009). This sort of biochemical plasticity, referred to as inducibility, may be a very important ecological and evolutionary plant trait, as it is a source of functional phenotypic variation in resistance and thus, potentially, a trait under selection (Agrawal et al., 2015). Considering a single plant genotype, inducibility is usually estimated as the difference between the induced vs. the constitutive concentration of PSM. In large plants, estimating inducibility of a PSM may require a repeated measures design within the same subject, i.e., before and after challenge with real insect herbivory (Morris et al., 2006). However, a common undesired side-effect is that sampling plant tissues may itself be an inducer of plant defenses, which would alter the estimation of inducibility (see Moreira et al., 2012, for example). Even more challenging can be the establishment of any relationship between inducibility of PSM and the effective resistance against real herbivory. This requires knowing the concentration of PSM before and after exposure of both constitutive and induced phenotypes to real herbivory when measuring plant damage or insect performance. For small plants and to evaluate plant traits that require destructive sampling, this problem can only be solved using clonal replicates of the same genotype.

At the intraspecific level, while genetic variation and plasticity of PSM are widely recognized, the ecology and evolution of PSM are far from being clear, and the role itself of PSM as traits providing direct effective resistance against herbivores is currently under debate (Carmona et al., 2011; Cárdenas et al., 2014). Despite the common finding of positive associations between concentration of PSM and resistance to specific herbivores (Borg-Karlson et al., 2006; Erbilgin et al., 2006; Kannaste et al., 2009; Zhao et al., 2011b), cause-effect studies demonstrating the direct role of particular PSM or blends of PSM on resistance are much less frequent (Agrawal, 2005; Iason et al., 2011).

In this study we tested the hypothesis that intraspecific variation in effective resistance of maritime pine (*Pinus pinaster*) to a key insect herbivore, the pine weevil (*Hylobius abietis*), may be predicted by variation in inducibility of PSM. We used clonally replicated genotypes with known family structure from 10 populations representative of the main distribution range of maritime pine. We performed an exhaustive characterization of constitutive concentrations and profiles of PSM in the stem phloem and their associated induced changes after application of methyl jasmonate (MJ), a plant hormone involved in signaling of chewing herbivore damage (Sampedro et al., 2011b; Schiebe et al., 2012; Zas et al., 2014). Both constitutive and MJ-induced clonal copies were exposed to insect herbivory to measure plant damage as a proxy of resistance (i.e., the greater the damage, the lower the resistance). Then we explored to what extent resistance to herbivory may be predicted by the constitutive concentration of PSM in the stem, and the role of its inducibility in predicting the increase in resistance once the plant is induced. Since the non-independence due to relatedness among genotypes, families and populations may affect the relationships between PSM and resistance, we accounted for the population structure and the relative kinship among individuals within populations.

## Materials and Methods

### Study System

We used *P. pinaster* as a model tree system, a Mediterranean conifer species with a fragmented distribution displaying large and structured among-population genetic variation (Vendramin et al., 1998; Bucci et al., 2007; Jaramillo-Correa et al., 2015). Populations largely differ in life history traits (Tapias et al., 2004; Grivet et al., 2013), including those putatively related to resistance to biotic stress and PSM (Sampedro et al., 2011b; Zas et al., 2015).

The pine weevil *H. abietis* is a bark-chewing insect herbivore with Palaearctic distribution, and is specialized in young conifers, feeding on several species (Leather et al., 1999). It harbors low neutral genetic variation across its large distribution range (Conord et al., 2006). The pine weevil is a harmful forest pest that causes severe damage on young conifer seedlings, and has a strong impact on plant fitness and early survival (Langström and Day, 2004; Zas et al., 2006, 2014). Host resistance has been showed to be related to plant survival and to be heritable (Zas et al., 2005). The pine weevil became a model insect for studies of resistance in pine trees (see for instance Nordlander et al., 2003; Wainhouse et al., 2005; Björklund, 2007; Nordlander et al., 2011; Sampedro et al., 2011b; Zas et al., 2014, 2017). The success in the regeneration of conifer forests is highly contingent on this insect (Langström and Day, 2004), which causes important economic losses in large part of Europe (Leather et al., 1999). Larval stages feed on stump roots of recently cut trees (Nordenhem and Nordlander, 1994), and newly emerged adults migrate in massive numbers to fresh clear-cuts in spring (Solbreck and Gyldberg, 1979; Tan et al., 2011). Generation times may vary from 1 to 3 years depending on the location in Europe (Day et al., 2004; Wainhouse et al., 2014). The longer generation times observed at higher latitudes, and the continuous masses of conifer forests, particularly in North Europe, strongly facilitate pine weevil dissemination, establishment, and subsequent damage among areas with clear-cuts for at least two up to four seasons after harvesting (Örlander et al., 1997). Apart from control and management measures to reduce the impact of the insect on plant fitness and survival (Leather et al., 1999; Nordlander et al., 2011), it is important to know the defensive potential in conifers to deal with this pernicious pest.

### Plant and Herbivore Material

**FIGURE 1:**
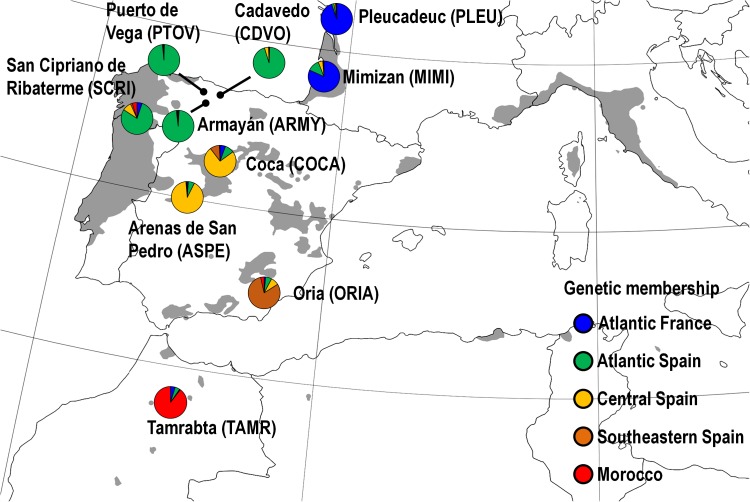
Natural distribution range of maritime pine (shaded area) and location of the ten studied populations (colored circles). Circle color represents the genetic membership of the populations based on STRUCTURE (see Supplementary Methods SM2). Populations were coded with four capital letters (in brackets). Modified from EUFORGEN (2009).

Ten maritime pine populations belonging to the collection ‘CLONAPIN Bank 1’ were selected along a latitudinal gradient across the natural distribution range of the species (Figure 1), from the Atlantic France to North Africa. Briefly, open-pollinated seeds were collected from five mother trees randomly selected in natural stands within each of the ten populations, resulting in fifty half-sib families (i.e., known ‘mother’ and unknown ‘father’). A separation of at least 50 m between selected mother trees was performed to minimize inbreeding (González-Martínez et al., 2006). Five seeds from each mother tree were sown and cultured in the facilities of SERIDA (Asturias, Spain), producing the complete clonal ortet collection, which comprised 250 genotypes (10 populations × 5 families × 5 half-sib genotypes). These genotypes were then clonally propagated by recurrent early cutting as described in Majada et al. (2011). A total of 205 out of the 250 original genotypes (belonging to 48 families out of the original 50) were eventually included due to low germination rate and/or rooting efficiency.

The cuttings were grown at SERIDA Agricultural Station (Grado, Asturias, Spain) in 200 mL containers containing peat and perlite (70:30 v/v), then transported to Norfor Forest Nursery (Figueirido, Pontevedra, Spain) and transplanted to 2 L pots. In September 2012, when pine cuttings were around 2-years old, the plants were transferred to an environmentally controlled greenhouse in the MBG-CSIC (Pontevedra, Spain). Plants were fertilized with a slow release fertilizer (Granum, Soaga SL, Vilanova de Arousa, Spain, NPK 11-22-9), supplemented with a foliar fertilizer (Fertimón, Progando SL, Galicia, Spain), and watered twice a week. Pines were allowed to acclimatize until the start of the experiment by December 2012. During this period, mean temperatures and relative humidity were recorded during the day (25.6 ± 0.06°C and 57.2 ± 0.08%, respectively) and during the night (18.2 ± 0.05°C and 69.0 ± 0.05%, respectively).

Adult weevils were collected at a clear-felled pine forest in Monte Castrove (Pontevedra, Spain, 42°2702300N, 8°4301900W) using Nordlander traps (Nordlander, 1987) and maintained in culture chambers at 10°C several weeks until the start of the herbivory bioassays (see below). During that period, the pine weevils were fed weekly with fresh twigs of maritime pine.

### Experimental Design

Plants were assigned to treatments following a factorial split-plot design replicated in 5 blocks, with population as the whole plot factor (ten populations) and the factorial combination of family (3–5 families per population) and the MJ induction treatment (2 levels: control and induced) as the split factor. Two clonal replicates of each genotype from each family were included in each of the two MJ-induction treatments. One of these copies was used for chemical analyses and the other for evaluation of the effective resistance against the pine weevil. The experiment comprised 814 plants from 10 populations, with 3–5 families per population, 5 half-sibs (genotypes) per family, and 4 clonal replicates per genotype (two of them, control and MJ, used for chemical analysis; and two, control and MJ, used in the weevil bioassays). A detailed graphical scheme of the experimental design can be found in the Supplementary Figure SM1.

### Induction Treatment

Plasticity of PSM in response to biotic stress has been extensively studied in maritime pine. Large changes in the concentration and chemical profile of total or specific PSM using either the exogenous application of MJ (Moreira et al., 2009), real herbivory (Moreira et al., 2013a), or both (Sampedro et al., 2011b; Moreira et al., 2012) have been reported in this and other pine species (Moreira et al., 2014; Carrillo-Gavilán et al., 2015). In December 2012, plants allotted to induction were transferred to a separate chamber prior to treatment application to avoid cross-contamination with control plants. Then, MJ-induced plants were sprayed with a suspension of 25 mM of 95% MJ (Sigma–Aldrich, #39270-7) in deionized water with 2.5% ethanol (v/v). Control plants were treated just with the carrier solution. Both solutions were sprayed over the aboveground part of each plant to runoff (∼10 mL per plant). MJ concentration and time elapsed between MJ application and sampling (1 month, see below) were based on previous studies (Moreira et al., 2013b; Zas et al., 2014). This lapse of time allowed plants to develop induced and long-lasting anatomical structures (i.e., resin ducts and polyphenolic parenchyma cells) that are involved in the production of induced defensive PSM (Moreira et al., 2015).

### Plant Sampling, Measurements and Analysis of Plant Secondary Metabolites

One month after MJ-application, two of the clonal copies of each genotype (one treated with MJ and one control) were destructively harvested by blocks, cutting the stem aboveground. Needles were separated from the stem and two 2-cm-long stem subsamples were immediately collected from its middle part, flash-frozen in liquid nitrogen, and stored at -80°C. We then analyzed the composition and concentration of PSM in 3 out of the 5 blocks (260 plants). Briefly, terpenoids were extracted from one of the frozen stem subsamples with HPLC-grade hexane (HiPersolv Chromanorm #83992.320) and separated, identified and quantified by gas chromatography-mass spectrometry (GC-MS) and GC-flame ionization detection (GC-FID). Phenolics were extracted from the other stem subsample with HPLC-grade methanol (HiPersolv Chromanorm #152506X) and separated, identified and quantified by ultra high performance liquid chromatography-mass spectrometry (UHPLC-MS) and UHPLC-diode array detection (UHPLC-DAD). A detailed description of the analytical procedures and data processing prior to statistical analyses can be found in the Supplementary Methods SM1).

We identified 118 PSM that were classified into eight chemical groups [monoterpenes, sesquiterpenes, diterpenes, flavonoids, hydroxycinnamic acids (HCAs), lignans, eugenols, and fatty acids]. For the purpose of the current study, we only considered those compounds present in at least 5 individuals of the control or MJ-induced plants for each population. Ninety-eight chemical compounds were finally included in the eight chemical groups (Supplementary Tables SM1, SM2).

### Herbivory Bioassays

At the time plants not subjected to herbivory were harvested for chemical analyses, the remaining two clonal replicates (one control and one treated with MJ) were used for evaluating the resistance of each genotype to the pine weevil, following López-Goldar et al. (2016), with modifications. Briefly, a transparent plastic tube was fitted around each plant and a randomly selected pair of weevils (one male and one female) were put on each plant and allowed to feed along the entire plant for 48 h. A fine-mesh was fitted at the top of the tube to prevent weevil escape. Weevils were starved in a Petri dish with moist filter paper (48 h at 15°C in dark) and pre-weighed prior the herbivory treatment. After 48 h of exposure to herbivory along the entire plant, weevils were removed and the damage caused by the weevils in the stem of each plant was measured at the nearest mm^2^ using calibrated area templates.

### Genetic Variation in Resistance to Herbivory

Variation in the damage caused by *H. abietis* in the stem was analyzed by fitting a mixed model using the PROC MIXED procedure in SAS v9.4 (Littell et al., 2006), with the induction treatment (MJ), population (P), family within population [F(P)] and MJ × P and MJ × F(P) interactions as fixed factors. The MJ × P and MJ × F(P) interactions are informative of the variation in the responses to the induction treatment among populations and families within populations, respectively. Block (B), the B × P interaction and the genotype [MJ × F(P) × B] were considered random factors. The B × P interaction was considered a random factor in order to analyze the main effect of P with the appropriate error term in the split-plot design (Littell et al., 2006). The genotype was also included as a random factor to account for the dependence between the two clonal replicates subjected to herbivory within each block. Damage was square root-transformed to achieve normality. Heterogeneous residual variance models across P were fitted. Weevil weight, as a proxy of weevil size, and stem diameter of the plant, both known to affect feeding rate (Wainhouse et al., 2004; Wainhouse et al., 2005), were included as covariates because they improved model fit. The same model was fit to explore the genetic variation among genetic groups of populations (GP) and within GP (families within each genetic group). Genetic groups of populations were identified and characterized elsewhere using molecular markers (Jaramillo-Correa et al., 2015; Serra-Varela et al., 2015), and corresponded to: Atlantic France (PLEU and MIMI), Atlantic Iberia (PTOV, CDVO, ARMY and SCRI), Central Iberia (COCA, ASPE), Southwestern Iberia (ORIA) and Morocco (TAMR) (Figure 1).

In order to quantify the relative contribution of each factor to the genetic variation among populations and families within populations in resistance to the pine weevil, the previous mixed model was run again assuming all factors as random factors. Variance components of each factor were estimated by restricted maximum likelihood.

### Relationships Between PSM and Resistance to Herbivory

To explore the causal relationships between resistance and PSM in the stem of *P. pinaster*, we performed pairwise correlations and multiple regression analyses between the weevil damage and PSM concentration at the genotypic level. Exploring the implication of PSM in resistance to herbivory in individuals within populations across the main distribution range will allow us to predict which PSM or blend of PSM within a genotype might be the optimal to determine the highest resistance to herbivory in the entire species. The clonal structure of our pine collection allowed for this genotypic-based analysis.

Because genotypes belong to a hierarchical collection of families within populations and to different populations in maritime pine, we accounted for the non-independence among genotypes within families and populations to avoid genetic effects due to relatedness in the correlation analyses (see Supplementary Methods SM2). For that, we included the population structure (*Q*) and kinship (*K*) matrices at the genotypic level using a mixed-model approach as described in Yu et al. (2006). Briefly, *Q* and *K* matrices were constructed based on 126 SNPs: for the *Q* matrix, we performed a Bayesian cluster analysis in STRUCTURE v 2.3.4 (Pritchard et al., 2000), whereas for the *K* matrix we used SPAGeDi (Hardy and Vekemans, 2002) based upon Loiselle et al. (1995) kinship coefficients. The *Q* and *K* matrices were then incorporated to the mixed models in order to account for the multiple level of relatedness among individuals (see Supplementary Methods SM2).

In addition, we fitted a mixed model for constitutive (C) and for the inducibility (MJ – C) of each total and individual PSM and weevil damage. For each defensive mode, population (P) and family within population [F(P)] were considered as fixed factors. Block (B) and the B × P interaction were considered random factors as above. *Q* and *K* matrices were incorporated into each mixed model to account for the genetic relatedness among genotypes. The predicted values of the variables from each plant in each mixed model were then used for the pairwise correlations and multiple regression analyses. Because PSM data were available in 3 out of 5 blocks, pairwise correlations and multiple regression analyses with weevil damage were performed only in 102 genotypes. Given that we were not able to extract the predicted values for all 102 genotypes after implementing *Q* and *K* matrices, and also not all clonal copies of the same genotype were available, sample sizes were slightly lower than the original 102 genotypes for each defensive state.

Two types of evaluations were performed to explore the relationships between PSM and weevil damage in the stem of *P. pinaster*. First, to observe the basal resistance without interference of the induction treatment, we explored the relationships between constitutive (C) PSM and damage by *H. abietis*. Second, we explored the relationships between the estimated inducibility of each total and individual PSM (MJ – C) with the inducibility of resistance to the pine weevil after MJ application. For each type of analysis, Pearson correlations between the concentration of each total and individual PSM and weevil damage were performed using PROC CORR in SAS v9.4. To estimate all the pairwise correlations without inflating Type I error due to multiple testing, *p*-value adjustments were performed using the False Discovery Rate (FDR) for *p* < 0.05 (Benjamini and Hochberg, 1995). Also, for the same two evaluations (constitutive and inducibility), multiple regression analyses were performed with PROC REG in SAS v9.4 to explore to what extent the PSM blend predicted herbivory resistance using the stepwise model selection method on the 98 individual PSM. We applied a more stringent threshold than default parameters (α = 0.05; default, α = 0.15) when including and excluding variables during the stepwise method to reduce type I error due to the large number of PSM used. Predictor variables were previously standardized (mean = 0, variance = 1) and influential observations (measured with Cook’s *D*) that did not meet the requirements were excluded from the analysis. Normality of the residuals was achieved using square-root transformation of the response variable (weevil damage) when necessary.

## Results

### Sources of Variation in Resistance to Herbivory by *Hylobius abietis* in *Pinus pinaster*

**FIGURE 2:**
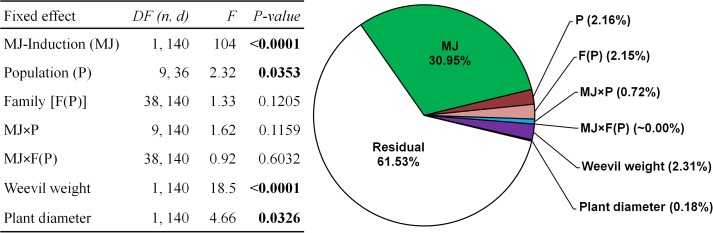
Summary of the mixed model testing the effects of the induction treatment with methyl-jasmonate (MJ), population (P), family within population [F (P)] and the MJ × P and MJ × F(P) interactions on the early resistance to herbivory by the pine weevil on plants from ten maritime pine populations. Variance components for each effect and the residual are shown in the companion pie chart. Weevil weight and plant diameter were included as covariates. Significant *p*-values (*p* < 0.05) are highlighted in bold. A total sample size of 205 genotypes was used.

**FIGURE 3:**
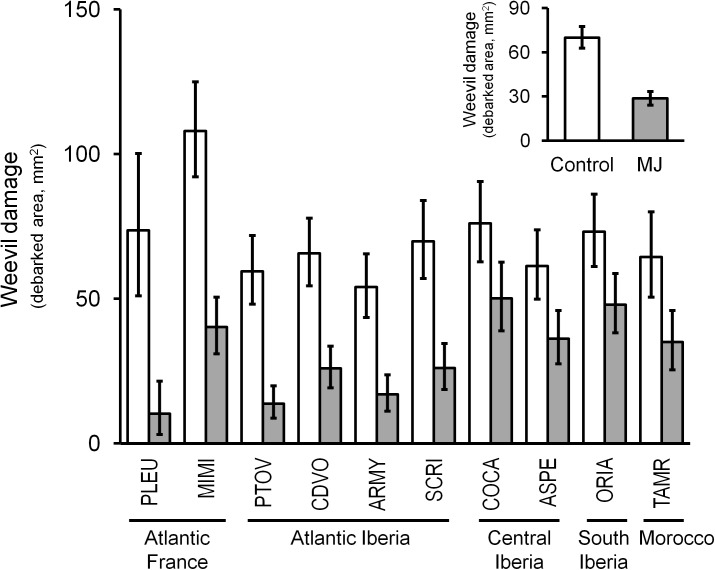
Intraspecific genetic variation in constitutive resistance (white bars) and MJ-induced resistance (gray bars) to the pine weevil across 10 maritime pine populations representing its main distribution range, grouped by the main genetic groups in the species. The inset panel shows the overall effect of the MJ-induction on the resistance to weevil herbivory in the species. Bars represent the least square mean ± SE (*N* = 8–25 plants for each population; *N* = 197–205 plants for each induction treatment in the small panel). A total sample size of 205 genotypes was used.

Resistance to weevil herbivory largely and significantly varied among populations but not between families within populations (companion table in Figure 2). The highest constitutive resistance was observed for the Spanish Atlantic population PTOV, whereas the most constitutively susceptible was the French Atlantic population MIMI (Figure 3). Large genetic variation was also found among genetic groups of populations (*F*_4,140_ = 4.73, *P* = 0.001), revealing similar patterns of constitutive resistance among populations of the same genetic group. The highest constitutive resistance was found for the Atlantic Spanish group (61.5 ± 7.3 mm^2^ of damage), whereas the Atlantic French group was the most susceptible (94.9 ± 12.3 mm^2^ of damage).

The MJ-induction treatment strongly increased resistance to herbivory in the stem of all populations (companion table in Figure 2), reducing the overall damage caused by the pine weevil to less than half compared to non-induced plants (Figure 3, inset panel). The French population PLEU showed the highest induced resistance, whereas COCA emerged as the less resistant after MJ application (Figure 3). The inducibility of resistance (i.e., the plasticity to MJ application) was similar for all populations and families [no significant MJ × P and MJ × F(P) interactions, companion table in Figure 2], but differed among genetic groups (MJ × GP interaction, *F*_4,140_ = 3.36, *P* = 0.012). Interestingly, greater inducibility of resistance after induction was found in both Atlantic genetic groups (71 and 67% in Atlantic France and Atlantic Spain, respectively) than in the other genetic groups (Central Spain, 37%; Southeastern Spain, 34%; and Morocco, 45%).

The main effects in our experimental design, namely MJ-induction, population, family and their interactions explained 36% of the total variance in resistance. MJ-induction was by far the most relevant factor, absorbing most of the explained variation in resistance among the studied effects (>80% of the explained variance), with the contribution of all remaining factors being comparatively much lower (Figure 2).

### Concentration of PSM in the Stem as Predictor of Damage by the Pine Weevil at the Genotypic Level

We performed pairwise correlations in order to explore the relationship between concentration of total and individual PSM in the stem with weevil damage, while accounting for population structure and genetic relatedness among individuals. Total and individual PSM did not show any significant negative relationship with weevil damage at the constitutive level (Table 1 and Supplementary Table SM3). When we explored the inducibility, total flavonoids and total lignans showed significant negative relationships with weevil damage (Table 1). Consistently, when considering individual PSM, significant negative correlations were found for the inducibility of two lignan hexoside derivatives, and marginally significant for three lignan xyloside derivatives and taxifolin derivative 1, after FDR correction (Supplementary Table SM3). As an exception, eugenol was the only PSM that was positively related with weevil damage at the constitutive level (Supplementary Table SM3).

**Table 1:** Pearson correlations between damage by the pine weevil, as a proxy of resistance, and the concentration of total plant secondary metabolites (PSM) in the stem phloem of 102 genotypes from 10 populations, representing the main distribution range of maritime pine.

Plant secondary metabolites	Constitutive (C) (*N* = 89)		Inducibility (MJ – C) (*N* = 96)	
		
	Pearson *r*	*P*-value	Pearson *r*	*P*-value
***Terpenes***				
Total monoterpenes	-0.05	0.610	0.08	0.410
Total sesquiterpenes	-0.13	0.240	0.12	0.253
Total volatile terpenes	-0.23	0.032	0.11	0.302
Total diterpenes	-0.01	0.946	0.17	0.087
***Phenolics***				
Total eugenols	0.17	0.114	-0.19	0.068
Total flavonoids	∼0.00	0.993	**-0.33**	**0.001**
Total hydroxycinnamic acids	-0.12	0.250	-0.24	0.017
Total lignans	-0.15	0.156	**-0.36**	**<0.001**


*Analyses were performed separately for constitutive total PSM (C), and for the inducibility of total PSM (MJ – C) after methyl-jasmonate induction. Significant correlations are highlighted in bold after adjustment for multiple testing correction using false discovery rate (FDR) for *p* ≤ 0.05 (Benjamini and Hochberg, 1995). Data used for correlations were obtained from mixed models accounting for population structure and relative kinship among individuals. Genotypic sample size of each defensive state is indicated in brackets. Due to the fact that some clonal replicates (control or MJ-induced) were not available for several genotypes, data points in the inducibility dataset were slightly lower than the original number of genotypes used.*

We used multiple regression analyses looking for the relative predictive value of individual PSM in the relationship between PSM and damage by *H. abietis* in the stem, while accounting for population structure and relative kinship among genotypes in *P. pinaster*. At the constitutive level, 18 individual PSM (10 terpenes and 8 phenolics; Table 2) significantly predicted 70% of the total variation in constitutive weevil damage (*F*_14,66_ = 18.97, *P* < 0.001). Six PSM (4 terpenes and 2 phenolics) were negatively related with weevil damage, as shown by their negative standardized regression coefficients (Table 2). With regard to inducibility, 12 PSM (8 terpenes, 3 phenolics and 1 fatty acid; Table 2) significantly predicted 68% of the total variance of weevil damage (*F*_11,83_ = 17.32, *P* < 0.001). Eight PSM (6 terpenes, 2 phenolics) showed standardized regression coefficients that were negatively related to the variation in weevil damage after MJ-induction (Table 2).

**Table 2:** Summary of the multiple regression analysis explaining the resistance to the pine weevil using the concentration of individual PSM as predictor variables in constitutive (upper part of the table) and in inducibility (lower part of the table) defensive modes in 102 genotypes from 10 maritime pine populations.

Defensive mode	Plant secondary chemicals	β	Partial *R*^2^
**Constitutive PSM**	**β-Phellandrene + Limonene**	**-0.98**	**0.150**
*(N = 87)*	**Lignan hexoside derivative 1**	**-1.58**	**0.077**
Intercept = 7.908	**Ferulic acid**	**-0.33**	**0.028**
Model adjusted *R*^2^ = 0.70	**α-Copaene**	**-1.89**	**0.025**
*F*_18,62_ = 18.97	**Unk P11**	**-0.35**	**0.024**
*P* < 0.001	**α-Cubebene + α-Longipinene**	**-0.76**	**0.016**
	Coumaric acid hexoside	0.60	0.079
	Methyl thymyl ether	0.57	0.054
	α-Phellandrene	0.52	0.040
	Methyl eugenol	0.87	0.035
	Elemol	0.75	0.031
	Eugenol	0.36	0.029
	Bicyclosesquiphellandrene	1.31	0.028
	Myrcene	0.56	0.022
	Lignan xyloside derivative 2	0.40	0.021
	Citronellyl propionate	0.46	0.018
	Sabinene	0.53	0.015
	Unk P6	0.39	0.011
**Inducibility of PSM**	**Unk P5**	**-22.46**	**0.234**
*(N = 93)*	**Lignan hexoside derivative 2**	**-19.17**	**0.060**
Intercept = -36.03	**Isopimaric acid**	**-10.05**	**0.060**
Model adjusted *R*^2^ = 0.68	**Elemol**	**-12.11**	**0.058**
*F*_12,78_ = 17.32	***cis*-β-Ocimene**	**-9.04**	**0.048**
*P* < 0.001	**Methyl thymyl ether**	**-10.36**	**0.040**
	**Pimaric acid**	**-11.76**	**0.029**
	***trans*-Pinocamphone**	**-10.15**	**0.022**
	Oleic acid C18:1	10.03	0.048
	Abietic acid	31.24	0.039
	Ferulic acid hexoside	8.03	0.024
	*trans*-β-Ocimene	4.78	0.019


*The regression coefficients (*β*) and partial *R*^2^ of PSM included in the model after stepwise selection method are shown. PSM associated with resistance to weevil damage (negative *β*) are typed in bold. Linear regression model at each defensive mode is significant at *p* < 0.05. Genotypic sample size of each defensive state is indicated in brackets. Due to the fact that some clonal replicates (control or MJ-induced) were not available for several genotypes, sample size for the inducibility dataset was slightly lower than the original number of genotypes used. Unk P#, unidentified phenolic compound.*

## Discussion

In our study, large and structured genetic variation in resistance to herbivory by *H. abietis* in the stem was found among *P. pinaster* populations, as well as significant differences among genetic groups of populations in resistance after MJ-induction. In addition, the resistance patterns were strongly predicted by the multivariate concentration and profile of PSM of genotypes after accounting for the genetic relatedness, rather than by bivariate correlations with weevil damage. Furthermore, differences in the resistance patterns between the constitutive and inducibility strategies were observed. While a high proportion of the variance for weevil damage was explained by the constitutive PSM, the directionality of the pattern (i.e., resistance or susceptibility) was unclear. Conversely, inducibility of PSM, besides explaining also a large proportion of the variance in weevil damage, predicted greater inducibility of resistance mainly due to relevant plastic and specific changes in the profile and increases in the concentration of the chemical compounds after induction.

### Inter- but Not Intrapopulation Genetic Variation and Increased Resistance to Herbivory After Induction

Our results show that *P. pinaster* populations are variable in resistance against the bark chewing herbivore *H. abietis*, but we found no evidence of variation between families within populations. Using the same clonal collection, Elvira-Recuenco et al. (2014) reported similar results for intraspecific variation in constitutive resistance against the fungal pathogen *Fusarium circinatum*. Atlantic populations showed greater constitutive resistance (with MIMI population as the exception in our study) than those from the Mediterranean Basin in both studies. This was, perhaps, an unexpected outcome, given the wide taxonomic dissimilarity of the two enemies. *F. circinatum* is considered an alien invasive pathogen in Europe (Wingfield et al., 2008) where, to date, it has been detected in nature in only one adult *P. pinaster* plantation in the northern Iberian Peninsula (Iturritxa et al., 2013). These results suggest that similar functional defensive traits may be effective against both biotic enemies, and that populations from growth-prone environments are better defended against these biotic agents at early stages. Similarly, we found extensive genetic variation among genetic groups of populations. This suggests that the differences in resistance levels among individual populations might be due to greater similarity among genetically closely related populations than distantly related ones. We did not find, however, genetic variation among families within populations for resistance, which is contrasting with previous results using the same pine species (Zas et al., 2005; Elvira-Recuenco et al., 2014). This finding might be due to the strong effect of the induction treatment, which may have overwhelmed most of the variation between families, or likely to sample size, that might be too low for detecting family variation.

Boosting MJ signaling pathways by exogenous application of MJ to produce a phenotype with increased expression of chemical and physical defenses is a well-known experimental tool in conifers (Heijari et al., 2005; Zhao et al., 2011a; Moreira et al., 2013b; Zas et al., 2014) and was confirmed in this study, with MJ being by far the most relevant factor (>80% of the total explained variance in resistance). The lack of a significant MJ by population interaction suggests that the patterns of resistance to herbivory elicited by MJ are highly regulated and conserved in *P. pinaster*, and probably derived from common ancestral defensive mechanisms shared in the conifer lineage (Hudgins et al., 2004). Results from previous studies that explored the genetic variation in the inducibility of resistance to insect herbivores after MJ application reported contrasting results. MJ induction reduced herbivory damage in *P. radiata* (Moreira et al., 2013b) and *P. pinaster* (Sampedro et al., 2011b), but family genetic variation in the inducibility of resistance was only detected for the folivore *Thaumetopoea pityocampa* and not for *H. abietis* (Sampedro et al., 2011b; Moreira et al., 2013b), suggesting that resistance mechanisms would be differentially regulated within populations depending on the herbivore identity and/or the target tissue. Despite that we did not find a MJ × population interaction, we did find it out among genetic groups of populations, indicating that the inducibility of resistance after MJ application is genetically structured in the species, i.e., resistance plasticity is more similar among genetically closely related populations than among distantly related ones. Our results highlight the evolutionary relevance and adaptive value of induced defenses and inducibility as putative key traits explaining variation in resistance. In addition, our findings are in agreement with the predictions regarding the intraspecific patterns of variation in plant defense (Hahn and Maron, 2016), although more profound analyses are required to appropriately address the postulates of the theory, which are out of scope in this study.

### Accounting for Biases in Relationships Between PSM and Resistance Across and Within Species

While associations between concentration of PSM and resistance have been widely reported in conifers (Borg-Karlson et al., 2006; Erbilgin et al., 2006; Kannaste et al., 2009), cause-effect studies directly exploring the particular role of individual PSM or PSM blends with resistance have been little explored (Agrawal, 2005; Iason et al., 2011). Moreover, recent studies addressing the implication of PSM in resistance to herbivory across and within species show contrasting results (Moles et al., 2011; Rasmann and Agrawal, 2011; Abdala-Roberts et al., 2016; Moreira et al., 2018), putting the defensive role of PSM under strong criticism (Carmona et al., 2011; Cárdenas et al., 2014). Such controversy might have arisen due to, among other factors, a lack of control for genetic relatedness among species, populations and individuals, and the consideration of broad groups of PSM (e.g., all terpenoids or all phenolic) instead of small chemical groups or individual PSM. First, in order to explore the relationships between PSM and resistance, the genetic relatedness across or within species must be accounted for to avoid spurious trait-trait or trait-environment relationships. In this sense, phylogenetic correction allows to account for the variation in traits due to common ancestry across related species (Freckleton et al., 2002), whereas the implementation of population structure and relative kinship among individuals allow to account for the variation due genetic relatedness among populations and among individuals within families in a single species (Yu et al., 2006). Second, the role of PSM can be better and precisely described when explored in small chemical groups or at the individual scale, because exploring relationships only among broad classes of compounds with resistance might overstate the implication of some individual PSM from those large groups that are not adaptively configured for defense (Hamilton et al., 2001). Furthermore, previous studies that criticize the adaptive value of PSM against resistance to herbivory (Carmona et al., 2011; Cárdenas et al., 2014) explored not only the effect of a small fraction of the whole diversity of chemical compounds (mostly few types and broad groups of phenolics), but also overlooked the inducibility of PSM as a relevant trait associated with resistance. In this sense, calling into question the role of PSM in resistance to herbivory based only on the effect of concrete and broad groups of PSM and when their plasticity was not measured, may obscure the adaptive value of PSM due to biased overgeneralizations. An exhaustive characterization of the constitutive chemical profile and its inducibility of the individuals would be required to maximize the range of metabolites putatively related to resistance and to identify those that might accurately predict the variation in resistance across and within species.

### PSM as Resistance Markers Against Herbivory

Plant secondary metabolites have been found to play an essential role in plant resistance to herbivory in many and phylogenetically distant plant species (for instance, see Agrawal et al., 2002; Zhao et al., 2010; Rasmann et al., 2014; Mason et al., 2016); thus, pointing to their adaptive value (Futuyma and Agrawal, 2009; Agrawal, 2010). Developing novel and heritable defensive traits that allow to resist herbivore attacks is of particular relevance in long lived plants like trees (Futuyma and Agrawal, 2009).

In our study, i) we maximized the range of phenotypic variation in the concentration of PSM potentially related to resistance against herbivores by exploring the constitutive allocation to PSM and their inducibility in the stem, ii) minimized possible genetic biases by using clonal replicates, iii) and accounted for relatedness among genotypes within families and populations of *P. pinaster* during the analyses. Our results showed that inducibility of flavonoids and lignans were strongly related to the inducibility of resistance to the pine weevil when exploring pairwise relationships. Previous studies have suggested that flavonoids and lignans are involved in plant defense (see reviews in Treutter, 2006; Witzell and Martín, 2008), acting as direct defenses against biotic agents (Wallis et al., 2010; Villari et al., 2016) as precursors in cell wall lignification for physical defense (Ralph et al., 2006), and being involved in protection against oxidative stress (Treutter, 2006). Only the concentration of eugenol at the constitutive level was found to be positively related with *H. abietis* damage (i.e., negatively related to resistance), which contrasts with previous findings where it was found to be an antifeedant for the pine weevil (Borg-Karlson et al., 2006; Eriksson et al., 2008). It is noteworthy that those previous studies were performed under laboratory conditions using purified extracts of eugenol, thus overlooking the interactive effects with other chemical constituents in the PSM blend that would affect insect feeding behavior. An alternative explanation for this finding is that the effect of eugenol was observed to be short-lasting, with decreased anti-feedant properties after several hours of exposure (Borg-Karlson et al., 2006), maybe due to the high volatility of this PSM. It is possible that the very low constitutive concentration of eugenol had an attractant effect rather than repellent in higher concentrations. Surprisingly, we did not detect any relationship between the constitutive concentration of terpene subgroups or individual terpenes, and their inducibility, with resistance to the pine weevil, despite previous results supporting this link (Danielsson et al., 2008; Kannaste et al., 2009; Schiebe et al., 2012; Lundborg et al., 2016). Noteworthy, however, when we explored the whole phenotypic range, including both constitutive and MJ-induced clonal replicates, all total terpene subgroups, total eugenols and total lignans, and many individual terpenes and phenolics were strongly and positively related to resistance against herbivory in the stem (data not shown) as previously found in multiple studies (Porth et al., 2011; Sampedro et al., 2011b; Schiebe et al., 2012; Zas et al., 2014; Lundborg et al., 2016). This result, together with the lack of significant pairwise associations between constitutive PSM (and inducibility) and resistance, suggest that MJ may have artificially forced the pairwise relationships when exploring the whole phenotypic variation. Previous results reporting PSM-resistance relationships upon control and induced plants should thus be interpreted with care. Based on our findings, we suggest that the strong effect of MJ in the reduction of weevil damage is product of a large multivariate induction rather than smaller and punctual individual effects on specific PSM.

In fact, when we explored the predictive power of the whole PSM blend in the stem, comprising 98 chemical compounds of different chemical nature, on resistance to *H. abietis* damage, we found strong evidence that resistance is largely mediated by the concentration of a specific blend of PSM and also by changes in the chemical profile after induction. On the one hand, constitutive resistance was explained at the multivariate level by terpene volatiles (mono- and sesquiterpenes) and many simple phenolics or precursors of complex molecules (for example, eugenols; Dudareva et al., 2013). This indicates that undamaged plants might initially invest more in simple carbon-based compounds in the stem as a cost-saving strategy when the biotic risk of attack is expected to be low (Karban, 2011). Among these PSM, β–phellandrene + limonene and lignan hexoside 1 have been showed to be strong and effective herbivore repellent and antifeedant, respectively, in conifers and other tree species, with increased resistance found in plants with greater concentrations of these PSM (Schiebe et al., 2012; Villari et al., 2016). While β–phellandrene and limonene coeluted during the chromatographic run in our study, limonene was previously observed to be significantly induced by MJ, whereas β–phellandrene was not affected (Zhao et al., 2010) and seedlings with greater concentrations of limonene in the stem were less attacked by *H. abietis* (Danielsson et al., 2008). On the other hand, plasticity of PSM revealed an important allocation toward more complex PSM, and changes in the chemical profile of the PSM blend after MJ application. Induction by MJ strongly increased the investment in larger molecules in the stem, such as diterpene resin acids, which are known to be toxic and function as deterrents (Larsson et al., 1986; Tomlin et al., 1996), but also contribute to the sealing of wounded tissue and mechanically impede feeding activity (Ericsson et al., 1988; Phillips and Croteau, 1999). Two phenolics previously found in the pairwise correlations, Unk P5 and lignan hexoside derivative 2, predicted almost half of the variance in increased resistance relative to the whole inducible PSM blend, supporting prior work (Ralph et al., 2006; Villari et al., 2016), and suggesting that these compounds are probably key in resistance, needing thus further investigation.

Despite their general ecological function, the role of fatty acids in conifer resistance is not well understood. We found that the inducibility of oleic acid is associated with susceptibility to pine weevil in maritime pine, confirming that fatty acid composition may affect host suitability for insect pests (Ishangulyyeva et al., 2016). However, due to the important investment in resources that induced defenses usually imply (Cipollini and Heil, 2010), it is possible that the production of induced PSM comes, in part, from the utilization of fatty acids after remobilization and digestion of lipid bodies in the plastids (Bernard-Dagan, 1988).

Interestingly, abietic acid, one of the most well-known diterpene resin acids associated with resistance against insect herbivores (Wagner et al., 1983; Tomlin et al., 1996; Powell and Raffa, 1999), showed an opposite pattern in our study. Recent studies found, however, that gut microbiota of stem chewers and borers can help overcome host defenses, particularly diterpene resin acids, when they are present at relatively low concentrations (Boone et al., 2013; Berasategui et al., 2017). Indeed, such microbiota-mediated processes may confer advantages to such insect guilds (Fäldt, 2000 and references therein), a phenomenon that may well apply to *H. abietis* and thus needs to be examined further.

## Conclusion

Our study supports a large body of evidence accumulated over decades that host chemical responses are involved in resistance to herbivory at the intraspecific level. However, our study provides the first example of a comprehensive approach in which PSM inducibility in trees, and its role in resistance, are analyzed in a rigorous genetic framework that allows the exclusion of spurious associations due to among-subject relatedness. It follows that multivariate analyses of PSM, conducted in this way, provide more realistic information about the potentially causal relationships between PSM and resistance to herbivory than evaluating single pairwise relationships or using broad groups of compounds.

## Data Accessibility

The SNP dataset analyzed in this study is publicly available in the Zenodo repository (doi: 10.5281/zenodo.1445313).

## Author Contributions

LS and RZ designed the experiment, provided the reagents, performed the sampling, helped with the statistical and GC analyses and the interpretation of the results and improved the different versions of the manuscript. PB provided the infrastructure and know-how for the UHPLC analyses of phenolic compounds and helped with the interpretation of the associated data. CV helped with the UHPLC analyses of phenolic compounds and with the interpretation of the associated data. AB-K provided the infrastructure and background for GC-MS analyses of terpenoid compounds and helped with the interpretation of the associated data. DG developed the SNPs database, constructed the population structure and kinship matrices, and provided expertise on controlling for genetic relatedness. XL-G performed the sampling, all the chemical analyses, most of the statistical analyses, produced the results, wrote the first draft with RZ and LS, and lead on the improvement of subsequent versions of the manuscript. PB, CV, AB-K, and DG contributed equally to the final version of the manuscript.

## Conflict of Interest Statement

The authors declare that the research was conducted in the absence of any commercial or financial relationships that could be construed as a potential conflict of interest.
