# Supplementary material for: Inducibility of Plant Secondary Metabolites in the Stem Predicts Genetic Variation in Resistance Against a Key Insect Herbivore in Maritime Pine
Source: Front Plant Sci. 2018 Nov 21;9:1651. doi: 10.3389/fpls.2018.01651 (PMC6258960; doi:10.3389/fpls.2018.01651)
Supplement: Supplementary file 1 [file Table_1.docx]

Supplementary Material

Inducibility of plant secondary metabolites in the stem predicts genetic variation in resistance against a key insect herbivore in maritime pine

**Xosé López-Goldar*, Caterina Villari, Pierluigi Bonello, Anna Karin Borg-Karlson, Delphine Grivet, Rafael Zas, Luís Sampedro.**

*** Correspondence:** Corresponding Author: xlgoldar@mbg.csic.es

The following Supplementary Material is available for this article:

**1. Supplementary Data:**

**Methods SM1.** Extraction, identification and quantification, and data processing of plant secondary metabolites (PSM).

**Methods SM2.** Genotyping, construction and implementation of population structure (*Q*) and kinship (*K*) matrices in the mixed models using SNP data.

**2. Supplementary Tables:**

**Table SM1.** Identity of the 93 terpenoid compounds found in the bark of two years-old pine juveniles from ten natural populations of maritime pine by GC-MS analysis.

**Table SM2.** Identity of the 25 phenolic compounds found in the bark of ten natural populations of 2-years old maritime pine juveniles by UHPLC-DAD-MS analysis.

**Table SM3.** Pearson correlations between damage by the pine weevil and the constitutive concentration of individual plant secondary metabolites (PSM) and their inducibility in the stem phloem of 102 genotypes from ten populations representing the main distribution range of maritime pine.

**3. Supplementary Figures:**

**Figure SM1.** Graphical scheme of the experimental design.

**1. Supplementary Data**

**Methods SM1. Extraction, identification and quantification, and data processing of plant secondary metabolites (PSM).**

Only 3 out of 5 blocks (260 plants) were used for the chemical analysis.

One of the two flash-frozen stem subsamples from each individual plant was used for terpene analyses by gas chromatography-mass spectrometry (GC-MS) and gas chromatography-flame ionization detection (GC-FID), while the other stem subsample was utilized for phenolic analyses by ultra high performance liquid chromatography-mass spectrometry (UHPLC-MS) and UHPLC-diode array detection (UHPLC-DAD). Phloem was separated from the xylem by hand with a surgical knife on ice, cut into small pieces and ground in liquid nitrogen using mortar and pestle.

Extraction of terpenes was performed following Sampedro et al. (2010) with modifications. Briefly, ca. 300 mg (fresh weight) of the ground tissue was extracted for 24 h in 1 ml hexane (HiperSolv Chromanorm #83992.320) in an ultrasonic bath at 25ºC, using pentadecane (Sigma-Aldrich, #76510) as internal standard. The extract was then split into two vials, one of which was used directly for analysis of volatile terpenes (mono and sesquiterpenes) whereas the other was dried under a flow of N_2_ for the analysis of diterpene resin acids (hereafter 'resin acids'). The dried extract was diluted in methanol with heptadecanoic acid (Sigma-Aldrich, #H3500) as internal standard and tetramethylammonium hydroxide (TMAH, Sigma-Aldrich, #334901) was added as methylation agent in a proportion of 1:10 (TMAH:methanol, vol:vol). Volatile terpenes were identified and quantified by GC-MS, whereas resin acids (as their methyl esters) were identified by GC-MS and quantified by GC-FID. The pellet in the extraction vial was oven-dried and weighed, and results expressed in a dry weight basis.

Extraction of phenolics was performed as described in Villari et al. (2012), with modifications. Briefly, 100 mg fresh weight (fw) of the ground tissue was extracted twice (for 24 h each time) in 500 μL of methanol (HiperSolv Chromanorm #152506X) at 4 ºC in darkness, using resorcinol (Sigma-Aldrich, #398047) as internal standard. The extract was centrifuged at 16,000 rcf for 10 minutes between extraction steps, and the two supernatants were combined. The pellet in the extraction vial was oven-dried and weighed and results were expressed in a dry weight basis. The non-polar resins in the combined extract were precipitated by adding 500 μL of deionized water (Milli-Q), followed by centrifugation at 16,000 rcf for 10 minutes. The supernatant was then transferred to another vial and the resin pellet was discarded. 800 μL of the clean extract were concentrated 8 times in a vacuum concentrator (Savant SPD2010 'Speedvac Concentrator', Thermo Scientific) and stored at -20 ºC until analyzed.

Identification and quantification of volatile terpenes and resin acids

Identification and quantification of volatile terpenes were performed at KTH (Stockholm, Sweden) using a GC-MS in total ion count mode (TIC). The instrument used was a HP6890 GC equipped with a DB-5 capillary column (30 m, ID 0.25 mm, film thickness 0.25 μm, Agilent Technologies, CA, USA), coupled to a HP5973 mass spectrometer (Agilent Technologies, CA, USA) and using the G1701EA MSD ChemStation software (Agilent Technologies, CA, USA). A volume of 1 μl of each sample was injected in splitless mode, using Helium as carrier gas. The oven temperature program was set at 40 ºC for 2 min, followed by a first temperature rise of 4 ºC·min^-1^ up to 200 ºC, then by a second temperature ramp of 10 ºC·min^-1^ up to 250 and maintained at this final temperature for 5 min. The injector temperature was set at 250 ºC.

The identification of each peak in the chromatogram was performed by comparing the retention times and mass spectra to that of available known standards (all from Fluka, Chemie AG, Buchs, Switzerland), to those in the NIST and Wiley Mass Spectral Libraries included in the analytical software, and from the correspondence between the calculated Kovat's Index (KI_C_) from alkane series with those published in the literature (Adams, 2007; KI_L_) using the same column type (Table SM1).

Volatile compounds were quantified by using calibration curves prepared with seven available commercial authentic standards of those compounds present in the samples (Table SM1). For those compounds whose standard was not available, relative quantification was done using the generated calibration curves from authentic standards of related compounds (Table SM1). Internal standard was used instead if no related compounds were found. All calibration curves showed linear regressions with R^2^>0.9999. Data handling for each chromatogram was performed as follows: peak integration was enabled between minutes 8 and 40, with a minimum detectable peak area of 500.000 area units, and peak width of 0.045 to avoid the integration of noise spikes or badly integrate narrow peaks. Instrument calibration and consistency were evaluated every 40 samples by injecting a known concentration (100 μg/mL) of all terpene standards, check internal standard (50 μg/mL) and alkane series (~10 μg/mL) to ensure both peak signal variation and retention time shifts were under control during the same session. Variability of the calibration (measured as coefficient of variation) was below 12% for all standards.

Identification of resin acids were performed at KTH (Stockholm, Sweden) using the same GC-MS hardware and instrument parameters used for volatile terpenes. The oven program for resin acids was set at 152 ºC for 2 min, followed by a temperature ramp of 3 ºC·min^-1^ up to 260 ºC and maintained at this final temperature for 5 min. The identification of each peak in the chromatogram was performed by comparing the retention times and mass spectra to those to available known standards (Sigma-Aldrich), to the NIST and Wiley Mass Spectral Libraries included in the analytical software and from the correspondence between the calculated Kovat's Index (KI_C_) from alkane series with those published in the literature (Adams, 2007; KI_L_) using the same column type. Resin acids were quantified at Misión Biológica de Galicia (Pontevedra, Spain) using a GC-FID and TotalChrom Workstation v6.3.2 (Perkin Elmer, MA, USA) as analytical software. The instrument used was a Clarus 500 GC equipped with an Elite-5 capillary column (30 m, ID 0.25 mm, film thickness 0.25 μm, Perkin Elmer, MA, USA), coupled to a FID. Elite-5 column has identical chromatographic features as the DB-5 column used during the identification of resin acids. All instrument parameters were configured identically as for previous GC-MS analysis. Hydrogen was used as carrier gas. FID temperature was set at 300ºC. Quantification of all resin acids was performed by preparing a calibration curve of authentic standard of abietic acid (Sigma-Aldrich) (Table SM1). Individual compound concentration was expressed in mg·g^-1^ stem dry weight (dw). Data handling for each chromatogram was performed as follows: peak integration was enabled from minute 12 until the end of the run, with a minimum detectable peak area of 5.000 area units, and bunching factor of 1.0 to avoid the integration of noise spikes or badly integrate narrow peaks. Instrument calibration and consistency was evaluated as described for volatile terpenes, using abietic acid as external standard (100 μg/mL), check internal standard (50 μg/mL) and alkane series (μg/mL). Variability of the calibration (measured as coefficient of variation) was below 13% for all standards.

Identification and quantification of phenolic compounds by UHPLC-MS-DAD and UPLC-DAD

Identification and quantification of phenolic metabolites was performed following the procedure described by Raffa et al. (2017). Identification of phenolic metabolites was carried out at the Targeted Metabolomics Laboratory at the Ohio State University (Columbus, OH, USA) using a UHPLC 1290 (Agilent Technologies, CA, USA) coupled to a DAD 1260 (Agilent Technologies, CA, USA) in line with a hybrid Triple Quadrupole/Ion trap mass spectrometer 5500 (QTRAP, AB Sciex, MA, USA). DAD spectral data were recorded from 210 to 400 nm with phenolic compounds being detected at 280 nm. Mass spectrometry data were acquired in negative ion mode and processed using Analyst 1.6.1 software. The column used was an Acquity UPLC^®^ BEH C_18_ (50 x 2.1 mm ID, 130Å, 1.7 μm particle size, Waters, MA, USA). Sample and column temperatures were set to 24 ºC and 50 ºC, respectively. The binary mobile phase consisted of 0.1% acetic acid in water (solvent A), and 0.1% acetic acid in methanol (Solvent B), with a constant flow rate of 0.42 ml·min^-1^. The following linear gradient (cumulative run time (min), % solvent A) was used: 0.0, 93; 4.5, 85; 10.0, 70; 13.0, 10; 15.0, 0; 16.5, 0; 17.0, 93; 17.5, 93; 21.0, 93 (total run time 21 min). Phenolic compounds were identified by overlaying full scan mass chromatograms and DAD chromatogram traces at 280 nm to match the retention times of [M−H]^−^ parent ions to λ_max_ of individual compounds. Phenolic compounds were identified based on negative ion fragmentation pattern, congruence of λ_max_, retention time based on standards and on the literature (see Table SM2).

Quantification of phenolic compounds was performed at the Department of Plant Pathology, College of Food, Agricultural and Environmental Sciences at the Ohio State University (Columbus, OH, USA) using an Acquity UPLC^®^ H-class coupled to a DAD (Waters, MA, USA). DAD spectral data were recorded from 210 to 400 nm with phenolic compounds being detected and quantified at 280 nm using Empower v3.0 software (Waters, MA, USA). Column type, sample and column temperatures, solvents and linear solvent gradient were identical to those used in the identification phase. λ_max_ from DAD spectral data and retention time from UPLC^®^ were then compared with those obtained from the identification phase to assign the corresponding identity to the compounds when possible.

Calibration curves for phenolic quantification were prepared with eleven commercially available authentic standards (Apin, UK; Extrasynthése, France; and Sigma-Aldrich, Germany) related to those compounds present in the samples (Table SM2). For those identified compounds whose standard was not available, relative quantification was done using the generated calibration curves from authentic standards of closely-related compounds (Table SM2). If no related compounds were available, compounds were quantified as internal standard equivalents. All calibration curves showed linear regressions with R^2^ > 0.999. Those compounds that could not be tentatively identified were labelled as 'unknown' peaks (Unk P#) and quantified as internal standard equivalents (Table SM2). Individual compound concentration was expressed in mg·g^-1^ stem dw. In order to avoid processing noise spikes or badly integrated narrow peaks, the following processing thresholds were adopted: minimum detectable peak area of 10.000 area units and minimum peak width of 10 seconds. To ensure both peak signal variation and retention time shifts were consistent during the same session, instrument calibration was evaluated every 20 samples by injecting a known concentration (100 μg/mL) of all phenolic standards and check internal standard (50 ng/μL). Variability of the calibration (measured as coefficient of variation) was below 7% for all standards.

Data processing prior to statistical analyses

7 pairs and 1 trio of PSM were impossible to separate individually during the data handling and were treated as single compounds (Table SM1). From the selected compounds used for statistical analyses, individual PSM were summed up in their corresponding chemical groups as total PSM [total monoterpenes, total sesquiterpenes, total diterpenes, total flavonoids, total hydroxycinnamic acids (HCAs), total hydroxybenzoic acids (HBAs), total lignans, total eugenols, and total fatty acids]. The last two totals of PSM (eugenols and fatty acids) were found in the GC-MS analyses and were classified as phenolic compounds and fatty acids in Table SM1, respectively. Because the group of total hydroxybenzoic acids had only one PSM representative (Vanillic acid hexoside, Table SM2), it was not considered for the analysis of total PSM. Also, fatty acids were not included in the analyses of total PSM. Jasmonates were not included in the analyses because they were found only in the MJ-plants as a result of the application of the induction treatment.

**Methods SM2. Genotyping, construction and implementation of population structure (*Q*) and kinship (*K*) matrices in the mixed models using SNP data.**

Clonal replicates of the same individuals from the collection were genotyped for 200 single nucleotide polymorphisms (SNPs). SNPs were selected from two arrays developed in maritime pine (Chancerel et al., 2011; Plomion et al., 2016) that included 50 SNPs considered as neutral, 50 SNPs considered as adaptive based on previous studies, and 100 SNPs selected in genes involved in drought stress, wood formation, and responses to biotic stressors. DNA was extracted using the DNeasy Plant Mini Kit (Qiagen) from previously silicagel-dried needles of 220 individuals coming from the 10 populations (16-26 individuals per population). SNP genotyping was performed by the LGC Genomics using the KASPTM genotyping technology (<http://www.lgcgenomics.com>; LGC, Teddington, UK). A total of 126 SNPs and 214 genotypes were available for further analyses after SNP quality control (publicly available at Zenodo repository with doi:10.5281/zenodo.1445313).

In order to estimate whether the observed pattern of variation of constitutive and effective resistance is influenced by the genetic relatedness among genotypes within populations in the correlations between PSM and resistance to herbivory, we built a population structure matrix (*Q*) using the Bayesian cluster analysis available in STRUCTURE v. 2.3.4 (Pritchard et al., 2000) with the following parameters utilizing the SNP data: admixture model on correlated allele frequencies; burn-in of 10.000 steps followed by 100.000 iterations; number of clusters (*K*) set from 1 to 10; 6 runs were performed for each *K*. The number of genetic groups (*K*) for each marker was evaluated following Pritchard and Wen (2003) by plotting the mean L(*K*) and its variance over the runs for each *K* value using STRUCTURE HARVESTER (Earl and vonHoldt, 2011).

The optimal *K* plateaued at *K*=5, which was concordant with that from a study exploring a greater sample size of individuals, populations and SNPs (Jaramillo-Correa et al., 2015). This result suggests that the historical and demographic processes of the species is well reflected by the genetic signal obtained with the 126 SNPs, and consequently the *Q* and *K* matrices can be built using these SNPs. Similarity across runs with the same *K* was calculated with CLUMPP (Jakobsson and Rosenberg, 2007), and the resulting membership coefficients for each individual formed the *Q*-matrix.

The SNP dataset was used to build the kinship matrix (*K*), based on Loiselle et al. (1995) kinship coefficients, using SPAGeDi (Hardy and Vekemans, 2002). Negative coefficients indicated individuals that are less related than random individuals and were, thus, set to zero as in Yu et al. (2006).

Eight genotypes were removed prior the analyses because of inconsistencies in their *Q* and *K* data with their original assignment to population, family and genotype. In addition, four additional genotypes that were missing in the original design of the clonal collection of 250 genotypes were not included in the analyses. Hence, *Q* and *K* matrices finally comprise 202 genotypes and are publicly available at Zenodo repository (DOI: 10.5281/zenodo.1445313). Then, *Q* and *K* matrices can be incorporated to a mixed model as described below, following Yu et al. (2006):

y = X**β** + Q**v** + Z**u** + **e** (1)

Equation (1) shows an extended version of a mixed model. Above, **y** is a vector of phenotypic observations, X**β** represents those fixed effects other than the population structure, where **β** is a vector of fixed effects other than population group effects; Q is a matrix from STRUCTURE relating **y** to the vector of population effects **v**; Z**u** represents the random effects, where **u** is a vector of polygenic background effects; **e** is a vector of residual effects; and *X* and *Z* are incidence matrices of ones and zeroes relating **y** to **β**, and **u**, respectively. The variances of the random effects are assumed to be Var(**u**) = 2*KV_g_*, and Var(**e**) = *RV_R_*, where *K* is an *n × n* matrix of relative kinship coefficients that define the degree of genetic covariance between a pair of individuals; *R* is an *n × n* matrix in which the off-diagonal elements are 0 and the diagonal elements are the reciprocal of the number of observations for which each phenotypic data point was obtained; *V_g_* is the genetic variance; and *V_R_* is the residual variance (Yu et al., 2006).

**2. Supplementary Tables**

**Table SM1.** Identity of the 93 terpenoid compounds found in the bark of two year-old pine juveniles from ten natural populations of maritime pine by GC-MS analysis. Compounds are shown grouped by terpene chemical species and sorted by retention time (RT, in minutes). Compounds were identified by comparing the retention times and mass spectra to those from available known standards (Sigma-Aldrich), to the NIST and Wiley Mass Spectral Libraries and from the correspondence between the calculated Kovat's Index (KI_C_) with those published in the literature (Adams, 2007; KI_L_). Assignment of standard equivalent was based on similarity in retention time and chemical structure between the compound in the sample and the standard used for quantification. Compounds not present in ≥ 5 of the samples are underlined and were subsequently discarded from the data analyses.

| **Code** | **Chemical compound** | **RT (min)** | **KI_C_** | **KI_L_** |  | **Standard equivalent** |
| --- | --- | --- | --- | --- | --- | --- |
|  | |  |  |  |  |  |
| *Monoterpenes* | |  |  |  |  |  |
| M1 | Tricyclene | 9.783 | 921 | 921 |  | α-Pinene |
| M2 | α-Thujene | 9.985 | 926 | 924 |  | α-Pinene |
| M3 | α-Pinene | 10.270 | 934 | 932 |  | α-Pinene |
| M4 | Camphene | 10.754 | 947 | 946 |  | β-Pinene |
| M5 | Sabinene | 11.694 | 973 | 969 |  | β-Pinene |
| M6 | β-Pinene | 11.833 | 977 | 974 |  | β-Pinene |
| M7 | Myrcene | 12.370 | 991 | 988 |  | Limonene |
| M8 | α-Phellandrene | 12.833 | 1004 | 1002 |  | α-Terpinene |
| M9 | 3-Carene | 13.059 | 1010 | 1008 |  | α-Pinene |
| M10 | α-Terpinene | 13.295 | 1016 | 1014 |  | α-Terpinene |
| M11 | ρ-Cymene | 13.583 | 1024 | 1020 |  | α-Terpinene |
| M12 | Limonene | 13.760 | 1028 | 1024 |  | Limonene |
|  | β-Phellandrene | 13.760 | 1028 | 1025 |  |  |
| M13 | *cis*-β-Ocimene | 14.123 | 1038 | 1032 |  | Limonene |
| M14 | *trans*-β-Ocimene | 14.502 | 1048 | 1044 |  | Limonene |
| M15 | γ-Terpinene | 14.894 | 1059 | 1054 |  | α-Terpinene |
| M16 | α-Terpinolene | 16.005 | 1088 | 1086 |  | α-Terpinene |
| M17 | Linalool | 16.419 | 1099 | 1095 |  | Pentadecane |
| M18 | Solusterol | 16.627 | 1105 | 1102 |  | Pentadecane |
| M19 | α-Campholene aldehyde | 17.374 | 1125 | 1122 |  | Pentadecane |
| M20 | Norpinone | 17.748 | 1136 | 1135 |  | β-Pinene |
| M21 | *trans*-pinocarveol | 17.877 | 1139 | 1135 |  | β-Pinene |
|  | *cis*-Verbenol | 17.946 | 1141 | 1137 |  |  |
| M22 | *trans*-Verbenol | 18.085 | 1145 | 1140 |  | α-Pinene |
| M23 | exo-methyl Camphenilol | 18.214 | 1149 | 1145 |  | α-Pinene |
| M24 | *trans*-Pinocamphone | 18.639 | 1160 | 1158 |  | β-Pinene |
| M25 | Pinocarvone | 18.712 | 1162 | 1160 |  | β-Pinene |
| M26 | Borneol | 18.873 | 1167 | 1165 |  | α-Pinene |
| M27 | Terpinen-4-ol | 19.274 | 1178 | 1174 |  | α-Terpinene |
| M28 | α-Terpineol | 19.759 | 1191 | 1186 |  | α-Terpinene |
| M29 | Myrtenol | 19.968 | 1197 | 1194 |  | α-Pinene |
|  | Myrtenal | 19.975 | 1197 | 1195 |  |  |
| M30 | Nopol | 20.216 | 1204 | 1278 |  | α-Pinene |
| M31 | Verbenone | 20.379 | 1208 | 1204 |  | β-Pinene |
| M32 | *trans*-Carveol | 20.758 | 1219 | 1215 |  | Limonene |
| M33 | Cuminic aldehyde | 20.845 | 1222 | 1238 |  | Limonene |
| M34 | Methyl thymyl ether | 21.306 | 1235 | 1232 |  | α-Terpinene |
| M35 | Piperitone | 21.997 | 1254 | 1249 |  | Pentadecane |
|  | Linalyl acetate | 22.022 | 1256 | 1254 |  |  |
| M36 | Bornyl acetate | 23.100 | 1287 | 1284 |  | α-Pinene |
| M37 | *trans*-Pinocarvyl acetate | 23.560 | 1291 | 1298 |  | β-Pinene |
| M38 | ρ-Vinyl guaiacol ^P^ | 24.009 | 1314 | 1309 |  | Limonene |
|  |  |  |  |  |  |  |
| *Sesquiterpenes* | |  |  |  |  |  |
| S1 | α-Cubebene | 25.303 | 1353 | 1345 |  | Isolongifolene |
|  | α-Longipinene | 25.333 | 1355 | 1350 |  |  |
| S2 | Eugenol ^P^ | 25.455 | 1358 | 1356 |  | Pentadecane |
| S3 | Cyclosativene | 25.894 | 1372 | 1369 |  | Pentadecane |
| S4 | α-Ylangene | 26.011 | 1375 | 1373 |  | Isolongifolene |
| S5 | α-Copaene | 26.157 | 1380 | 1374 |  | Isolongifolene |
| S6 | Geranyl acetate ^ME^ | 26.279 | 1383 | 1379 |  | Pentadecane |
| S7 | β-Bourbonene | 26.458 | 1389 | 1387 |  | Pentadecane |
| S8 | β-Cubebene | 26.619 | 1394 | 1387 |  | Isolongifolene |
|  | Sativene | 26.640 | 1394 | 1390 |  |  |
|  | β-Elemene | 26.663 | 1395 | 1389 |  |  |
| S9 | Methyl eugenol ^P^ | 26.959 | 1404 | 1403 |  | Pentadecane |
| S10 | Longifolene | 27.153 | 1411 | 1407 |  | Isolongifolene |
| S11 | *trans*-β-Caryophyllene | 27.592 | 1425 | 1417 |  | β-Caryophyllene |
| S12 | β-Gurjunene | 27.871 | 1434 | 1431 |  | Isolongifolene |
| S13 | *trans*-Isoeugenol ^P^ | 28.407 | 1451 | 1448 |  | Pentadecane |
| S14 | α-Humulene | 28.642 | 1459 | 1452 |  | α-Humulene |
| S15 | α-Amorphene | 29.334 | 1481 | 1483 |  | Isolongifolene |
| S16 | Germacrene D | 29.525 | 1487 | 1484 |  | β-Caryophyllene |
|  | Phenethyl 2-methylbutyrate * | 29.554 | 1488 | 1486 |  |  |
| S17 | Phenethyl isovalerate * | 29.687 | 1493 | 1490 |  | Pentadecane |
| S18 | Bicyclosesquiphellandrene | 29.832 | 1497 | - |  | Isolongifolene |
| S19 | α-Muurolene | 30.048 | 1504 | 1500 |  | Isolongifolene |
| S20 | γ-Cadinene | 30.518 | 1520 | 1513 |  | Isolongifolene |
| S21 | δ-Cadinene | 30.739 | 1528 | 1522 |  | Isolongifolene |
| S22 | Zonarene | 30.816 | 1531 | 1528 |  | Isolongifolene |
| S23 | Cadina-1,4-diene | 31.018 | 1537 | 1533 |  | Isolongifolene |
| S24 | α-Cadinene | 31.173 | 1543 | 1537 |  | Isolongifolene |
| S25 | Elemol | 31.488 | 1553 | 1548 |  | Isolongifolene |
| S26 | Citronellyl propionate ^ME^ | 32.158 | 1576 | - |  | Pentadecane |
| S27 | Germacrene D-4-ol | 32.298 | 1581 | 1574 |  | β-Caryophyllene |
| S28 | Caryophyllene oxide | 32.531 | 1589 | 1582 |  | β-Caryophyllene |
| S29 | Longiborneol | 32.957 | 1604 | 1599 |  | β-Caryophyllene |
|  |  |  |  |  |  |  |
| *Diterpene resin acids and fatty acids* | |  |  |  |  |  |
| DT1 | Oleic acid C18:1 | 14.971 | 2105 | - |  | Heptadecanoic acid |
| DT2 | Pimaric acid | 19.161 | 2244 | 2237 |  | Abietic acid |
| DT3 | Sandaracopimaric acid | 19.707 | 2261 | 2256 |  | Abietic acid |
| DT4 | Unk DT1 | 20.623 | 2288 | - |  | Abietic acid |
| DT5 | Isopimaric acid | 21.156 | 2303 | 2297 |  | Abietic acid |
| DT6 | Levopimaric acid | 21.589 | 2314 | 2306 |  | Abietic acid |
|  | Palustric acid | 21.589 | 2314 | - |  |  |
| DT7 | Arachidic acid C20:0 | 22.281 | 2331 | - |  | Heptadecanoic acid |
|  | Unk DT2 | 22.281 | 2331 | - |  |  |
| DT8 | Dehydroabietic acid | 22.802 | 2344 | 2341 |  | Abietic acid |
| DT9 | 8,12-Abietadien-18-oic acid | 23.492 | 2362 | - |  | Abietic acid |
| DT10 | Abietic acid | 24.617 | 2390 | 2385 |  | Abietic acid |
| DT11 | Neoabietic acid | 26.882 | 2442 | 2443 |  | Abietic acid |
| DT12 | Behenic acid C22:0 | 31.057 | 2536 | - |  | Heptadecanoic acid |
|  |  |  |  |  |  |  |
| *Jasmonates* | |  |  |  |  |  |
| S30 | *trans*-Methyl jasmonate | 33.834 | 1635 | - |  | *trans*-Methyl jasmonate |
| S31 | *cis*-Methyl jasmonate | 34.265 | 1651 | 1648 |  | *cis*-Methyl jasmonate |
|  | α-Muurolol^✝^ | 34.265 | 1652 | 1644 |  |  |
| S32 | Methyl epijasmonate | 35.089 | 1680 | 1678 |  | Methyl epijasmonate |

Compounds were coded according to the chemical analysis group where they were detected during the runs. M#, S# and DT# refer to monoterpene, sesquiterpene and diterpene chemical analysis groups, respectively. Compounds with no code were considered co-eluted with the previous peak in the table and treated hereafter as a single compound in the data analysis. RT, retention time (min); KI_C_, calculated Kovat's Index using n-alkane series; KI_L_, Kovat's Index extracted from the literature (Adams, 2007) using the same column type, DB-5 (5%-phenyl methylpolysiloxane).

^✝^ α-Muurolol is a sesquiterpene, but coeluted with *cis*-methyl jasmonate during the GC-MS runs.

^P^ Aromatic compound (phenylpropanoid). ρ-Vinyl guaiacol, eugenol, methyl eugenol and *trans*-isoeugenol are phenolic compounds found in the GC-MS runs.

* Aromatic ester. Phenethyl 2-methylbutyrate and phenethyl isovalerate are not terpenes but are present as volatile compounds in pine species (Petrakis et al., 2005).

^ME^ Monoterpene ester.

**References:**

Adams, R.P. 2007. Identification of Essential oil components by Gas Chromatography/Mass Spectrometry. 4th Edition. Allured Publishing Corporation, Illinois, USA. 803 p.

Petrakis, P.V.; Roussis, V.; Papadimitriou, D.; Vagias, C.; Tsitsimpikou, C. 2005. The effect of terpenoid extracts from 15 pine species on the feeding behavoural sequence of the late instars of the pine processionary caterpillar *Thaumetopoea pityocampa*. Behavioural Processes, 69:303-322.

**Table SM2.** Identity of the 25 phenolic compounds found in the stem of ten natural populations of 2-year-old maritime pine juveniles by UHPLC-DAD-MS analysis. Compounds are sorted by retention time (RT, in minutes). Tentative identification was based on *m/z*, MS fragmentation and UV absorbance maxima compared with those obtained from literature or standards when available. Assignment of standard equivalent was based on similarity in retention time and chemical structure between the compound in the sample and the standard used for quantification.

| **Code** | **Assigned identity** | **RT (min)** | **UV λ max (nm)** | **[M-H]^-^(m/z)** | **Main ESI-MS fragments** | **References** | **Standard equivalent** |
| --- | --- | --- | --- | --- | --- | --- | --- |
| P1 | Vanillic acid hexoside | 1.867 | 253.4, sh 290.9 | 329 | 167, 108, 152 | c, f | Vanillic acid |
| P2 | Procyanidin trimer | 2.657 | 279.0 | 865 | 577, 407, 289, 425, 451, 125, 161 | d | Procyanidin B2 |
| P3 | Coumaric acid hexoside | 2.799 | 294.6 | 325 | 119, 163, 152 | d, e, f | Coumaric acid |
| P4 | Coumaroylquinic acid | 3.066 | 310.7 | 337 | 163, 119, 191, 93, 155, 173 | a, b, d | Coumaric acid |
| P5 | Taxifolin derivative 1 | 3.583 | 285.4 | 465 | 286, 276, 125, 177, 304, 153 | c, f | Taxifolin |
| P6 | Ferulic acid hexoside | 4.006 | 290.3, sh 316 | (711) 355 | 193, 134, 178, 149, 119 | e, f | Ferulic acid |
| P7 | Unk P1 | 4.152 | 291.5 |  |  |  | Resorcinol |
| P8 | Unk P2 | 4.434 | 280.5 |  |  |  | Resorcinol |
| P9 | Taxifolin derivative 2^S^ | 5.113 | 284.2 |  |  |  | Taxifolin |
| P10 | Unk P3 | 5.922 | 297.7 |  |  |  | Resorcinol |
| P11 | Lignan hexoside derivative 1 | 7.094 | 280.0 | 507 | 315, 327, 300, 345, 255 | c, f | Pinoresinol |
| P12 | Lignan hexoside derivative 2 | 7.388 | 280.0 | 507 | 315, 327, 300, 345, 255, 283 | c, f | Pinoresinol |
| P13 | Unk P4 | 7.436 | 287.8 |  |  |  | Resorcinol |
| P14 | Ferulic acid | 7.676 | 321, sh 218 |  |  |  | Ferulic acid |
| P15 | Unk P5 | 7.749 | 288.5 |  |  |  | Resorcinol |
| P16 | Lignan xyloside derivative 1 | 7.910 | 278.0 | 495 | 167, 179, 327, 146, 121, 315, 221, 345 | c, f | Pinoresinol |
| P17 | Lignan xyloside derivative 2 | 8.063 | 278.0 | 495 | 167, 179, 165, 327, 221, 146, 121 | c, f | Pinoresinol |
| P18 | Lignan xyloside derivative 3 | 8.113 | 278.0 | 495 | 167, 179, 327, 221, 146, 121 | c, f | Pinoresinol |
| P19 | Unk P6 | 9.092 | 278.0 |  |  |  | Resorcinol |
| P20 | Lignan deoxyhexoside | 9.637 | 280.0 | 491 | 315, 327, 300, 312 | c, f | Pinoresinol |
| P21 | Unk P7 | 10.447 | 263.3 |  |  |  | Resorcinol |
| P22 | Unk P8 | 12.758 | 263.3 |  |  |  | Pinoresinol |
| P23 | Unk P9 | 13.307 | 244.3 |  |  |  | Resorcinol |
| P24 | Unk P10 | 14.082 | 273.1 |  |  |  | Pinocembrin |
| P25 | Unk P11 | 14.287 | 277.4, sh 326.1 |  |  |  | Resorcinol |

sh: shoulder

S: tentative identity based on spectra.

Unk: unknown compound

References: a) Chen et al. (2011), J. Chem. Ecol.; b) Kammerer et al. (2004), Rapid. Comm. Mass. Spec.; c) Karonen et al. (2004), J. Agri. Food. Chem.; d) Lin and Harnly (2007), J. Agri. Food. Chem.; e) Pan and Lundgren (1996), Phytochem.; f) Wallis et al. (2011), For. Pathol.

**References:**

Chen, Y.; Whitehill, J.; Bonello, P.; Poland, T. 2011. Differential response in foliar chemistry of three ash species to emerald ash borer adult feeding. J. Chem. Ecol., 37: 29-39.

Kammerer, D.; Carle, R.; Schieber, A., 2004: Characterization of phenolic acids in black carrots (*Daucus carota* ssp *sativus* var. atrorubens Alef.) by high-performance liquid chromatography ⁄ electrospray ionization mass spectrometry. Rapid Comm. Mass Spec. 18, 1331–1340.

Karonen, M.; Hamalainen, M.; Nieminen, R.; Klika, K. D.; Loponen, J.; Ovcharenka, V. V.; Moilanen, E.; Pihlaja, K., 2004: Phenolic extractions from the bark of *Pinus sylvestris* L. and their effects on inflammatory mediators nitric oxide and prostaglandin E2. J. Agri. Food Chem. 52, 7532–7540.

Lin, L.; Harnly, J. 2007. A screening method for the identification of glycosylated flavonoids and other phenolic compounds using a standard analytical approach for all plant materials. J. Agric. Food Chem., 55: 1084-1096.

Pan, H.; Lundgren, L. N., 1996: Phenolics from the inner bark of *Pinus sylvestris*. Phytochemistry 42, 1185–1189.

Wallis, C., Eyles, A., Chorbadjian, R. A., Riedl, K., Schwartz, S., Hansen, R., Cipollini, D., Herms, D. A. and Bonello, P. 2011. Differential effects of nutrient availability on the secondary metabolism of Austrian pine (*Pinus nigra*) phloem and resistance to *Diplodia pinea*. Forest Pathology, 41: 52–58.

**Table SM3.** Pearson correlations between damage by the pine weevil, as a proxy of resistance, and the concentration of individual plant secondary metabolites (PSM) in the stem phloem of 102 genotypes from ten populations, representing the main distribution range of maritime pine. Analyses were performed separately for constitutive PSM (C), and for the inducibility of PSM (MJ – C) after methyl-jasmonate induction. Significant correlations are highlighted in bold after adjustment for multiple testing correction using false discovery rate (FDR) for p ≤ 0.05 (Benjamini and Hochberg, 1995). In brackets is the genotype sample size used for each analysis. Data used for correlations were obtained from mixed models accounting for population structure and relative kinship among individuals. Due to the fact that some clonal replicates (control or MJ-induced) were not available for several genotypes, data points in the inducibility dataset were slightly lower than the original number of genotypes used.

| Plant secondary metabolites | Constitutive (C)  *(N = 89)* | | Inducibility (MJ – C)  *(N = 96)* | |
| --- | --- | --- | --- | --- |
|  | *Pearson r* | *P-value* | *Pearson r* | *P-value* |
|  |  |  |  |  |
| *Monoterpenes* |  |  |  |  |
| Tricyclene | 0.06 | 0.595 | -0.01 | 0.907 |
| α-Pinene | -0.11 | 0.307 | 0.13 | 0.216 |
| Camphene | 0.07 | 0.505 | -0.03 | 0.775 |
| Sabinene | 0.09 | 0.396 | 0.13 | 0.199 |
| β-Pinene | -0.10 | 0.350 | -0.01 | 0.901 |
| Myrcene | 0.02 | 0.852 | 0.17 | 0.099 |
| α-Phellandrene | 0.06 | 0.598 | -0.13 | 0.200 |
| 3-Carene | 0.12 | 0.276 | 0.11 | 0.269 |
| α-Terpinene | 0.05 | 0.657 | 0.11 | 0.304 |
| ρ-Cymene | 0.09 | 0.396 | -0.08 | 0.450 |
| β-Phellandrene+Limonene | -0.21 | 0.048 | -0.07 | 0.522 |
| *cis*-β-Ocimene | -0.23 | 0.029 | -0.11 | 0.267 |
| *trans*-β-Ocimene | -0.13 | 0.229 | -0.02 | 0.818 |
| γ-Terpinene | 0.06 | 0.590 | 0.12 | 0.251 |
| α-Terpinolene | 0.07 | 0.492 | 0.12 | 0.246 |
| Linalool | 0.16 | 0.135 | -0.12 | 0.231 |
| Solusterol | 0.04 | 0.717 | 0.02 | 0.869 |
| α-Campholene aldehyde | 0.12 | 0.247 | 0.11 | 0.269 |
| Norpinone | 0.20 | 0.054 | 0.24 | 0.018 |
| *Trans*-pinocarveol+*cis*-Verbenol | 0.26 | 0.014 | 0.22 | 0.029 |
| *trans*-Verbenol | 0.07 | 0.515 | 0.15 | 0.140 |
| exo-methyl Camphenilol | - | - | 0.02 | 0.828 |
| *trans*-Pinocamphone | 0.34 | <0.001 | -0.12 | 0.238 |
| Pinocarvone | 0.19 | 0.081 | 0.19 | 0.066 |
| Borneol | 0.10 | 0.364 | -0.24 | 0.017 |
| Terpinen-4-ol | 0.20 | 0.056 | 0.14 | 0.186 |
| α-Terpineol | 0.06 | 0.601 | 0.20 | 0.049 |
| Myrtenal+Myrtenol | 0.25 | 0.016 | 0.19 | 0.064 |
| Verbenone | 0.06 | 0.546 | 0.06 | 0.564 |
| Methyl thymyl ether | -0.12 | 0.259 | -0.05 | 0.606 |
| Piperitone+Linalyl acetate | -0.21 | 0.049 | ~0.00 | 0.969 |
| Bornyl acetate | 0.08 | 0.464 | 0.16 | 0.125 |
| *trans*-Pinocarveyl acetate | 0.28 | 0.007 | -0.07 | 0.514 |
| Geranyl acetate | -0.10 | 0.339 | 0.02 | 0.879 |
| Citronellyl propionate | -0.04 | 0.726 | 0.04 | 0.713 |
| *Sesquiterpenes* |  |  |  |  |
| α-Cubebene+α-Longipinene | -0.15 | 0.163 | 0.05 | 0.650 |
| α-Ylangene | ~0.00 | 0.979 | 0.06 | 0.587 |
| α-Copaene | -0.16 | 0.136 | 0.10 | 0.319 |
| β-Cubebene+Sativene+β-Elemene | -0.23 | 0.028 | 0.02 | 0.812 |
| Longifolene | -0.03 | 0.793 | 0.07 | 0.523 |
| *trans*-β-Caryophyllene | -0.17 | 0.113 | 0.07 | 0.522 |
| β-Gurjunene | -0.22 | 0.038 | -0.01 | 0.945 |
| α-Humulene | -0.12 | 0.255 | 0.05 | 0.660 |
| α-Amorphene | -0.24 | 0.023 | 0.05 | 0.608 |
| Germacrene D+Phenethyl 2-methylbutyrate | -0.23 | 0.026 | 0.05 | 0.636 |
| Phenethyl isovalerate | -0.08 | 0.459 | 0.19 | 0.066 |
| Bicyclosesquiphellandrene | -0.16 | 0.138 | 0.07 | 0.482 |
| α-Muurolene | -0.08 | 0.438 | 0.10 | 0.313 |
| γ-Cadinene | -0.16 | 0.136 | 0.12 | 0.246 |
| δ-Cadinene | -0.22 | 0.040 | 0.05 | 0.640 |
| Zonarene | -0.19 | 0.069 | 0.03 | 0.797 |
| Cadina-1,4-diene | -0.22 | 0.037 | 0.02 | 0.853 |
| α-Cadinene | -0.19 | 0.068 | -0.02 | 0.829 |
| Elemol | 0.30 | 0.004 | -0.25 | 0.015 |
| Germacrene D-4-ol | -0.21 | 0.044 | 0.09 | 0.381 |
| Caryophyllene oxide | 0.16 | 0.136 | 0.25 | 0.015 |
| Longiborneol | -0.01 | 0.937 | 0.05 | 0.604 |
| *Diterpenes* |  |  |  |  |
| Pimaric acid | -0.07 | 0.541 | 0.24 | 0.019 |
| Sandaracopimaric acid | -0.07 | 0.523 | 0.21 | 0.034 |
| Unk DT1 | -0.18 | 0.097 | 0.15 | 0.154 |
| Isopimaric acid | 0.11 | 0.322 | -0.06 | 0.529 |
| Levopimaric+Palustric acids | 0.03 | 0.767 | 0.10 | 0.314 |
| Dehydroabietic acid | 0.10 | 0.350 | 0.25 | 0.014 |
| 8,12-abietadien-18-oic acid | ~0.00 | 0.979 | 0.15 | 0.148 |
| Abietic acid | -0.03 | 0.759 | 0.17 | 0.095 |
| Neoabietic acid | -0.07 | 0.496 | 0.12 | 0.238 |
| *Phenolics* |  |  |  |  |
| Vanillic acid hexoside | -0.04 | 0.692 | -0.23 | 0.026 |
| Procyanidin dimer | -0.04 | 0.677 | **-0.32** | **0.002** |
| Coumaric acid hexoside | -0.05 | 0.652 | -0.17 | 0.094 |
| Coumaroylquinic acid | 0.08 | 0.449 | -0.25 | 0.015 |
| Taxifolin derivative 1 | ~0.00 | 0.985 | -0.27 | 0.008 |
| Ferulic acid hexoside | -0.19 | 0.076 | -0.26 | 0.009 |
| Unk P1 | -0.12 | 0.251 | -0.07 | 0.479 |
| Unk P2 | -0.07 | 0.504 | -0.19 | 0.067 |
| Taxifolin derivative 2 | 0.11 | 0.316 | -0.10 | 0.346 |
| Unk P3 | -0.11 | 0.290 | -0.05 | 0.642 |
| Lignan hexoside derivative 1 | -0.20 | 0.059 | **-0.30** | **0.003** |
| Lignan hexoside derivative 2 | -0.05 | 0.641 | **-0.33** | **0.001** |
| Unk P4 | -0.07 | 0.500 | -0.08 | 0.422 |
| Ferulic acid | -0.13 | 0.218 | 0.03 | 0.736 |
| Unk P5 | -0.18 | 0.088 | **-0.45** | **<0.001** |
| Lignan xyloside derivative 1 | -0.09 | 0.421 | -0.26 | 0.010 |
| Lignan xyloside derivative 2 | -0.08 | 0.476 | -0.28 | 0.006 |
| Lignan xyloside derivative 3 | 0.10 | 0.345 | -0.27 | 0.008 |
| Unk P6 | -0.13 | 0.207 | **-0.30** | **0.002** |
| Lignan deoxyhexoside | -0.10 | 0.336 | -0.20 | 0.044 |
| Unk P7 | 0.08 | 0.438 | -0.11 | 0.274 |
| Unk P8 | -0.04 | 0.698 | -0.03 | 0.765 |
| Unk P9 | -0.02 | 0.832 | -0.05 | 0.621 |
| Unk P10 | -0.09 | 0.396 | -0.05 | 0.605 |
| Unk P11 | 0.08 | 0.455 | -0.02 | 0.881 |
| ρ-Vinyl guaiacol | -0.02 | 0.839 | 0.07 | 0.493 |
| Eugenol | **0.36** | **<0.001** | -0.12 | 0.225 |
| Methyl eugenol | 0.11 | 0.320 | -0.17 | 0.087 |
| *trans*-Isoeugenol | 0.10 | 0.365 | -0.05 | 0.608 |
| *Fatty acids* |  |  |  |  |
| Oleic acid C18:1 | 0.24 | 0.023 | -0.11 | 0.290 |
| Unk DT2+Arachidic acid C20:0 | -0.06 | 0.565 | 0.08 | 0.424 |
| Behenic acid C22:0 | 0.07 | 0.544 | -0.08 | 0.449 |

Unk: unidentified compound.

Dashes represent PSM that could not be estimated or not detected during chromatographic analyses at the corresponding defensive mode.

**3. Supplementary Figures:**


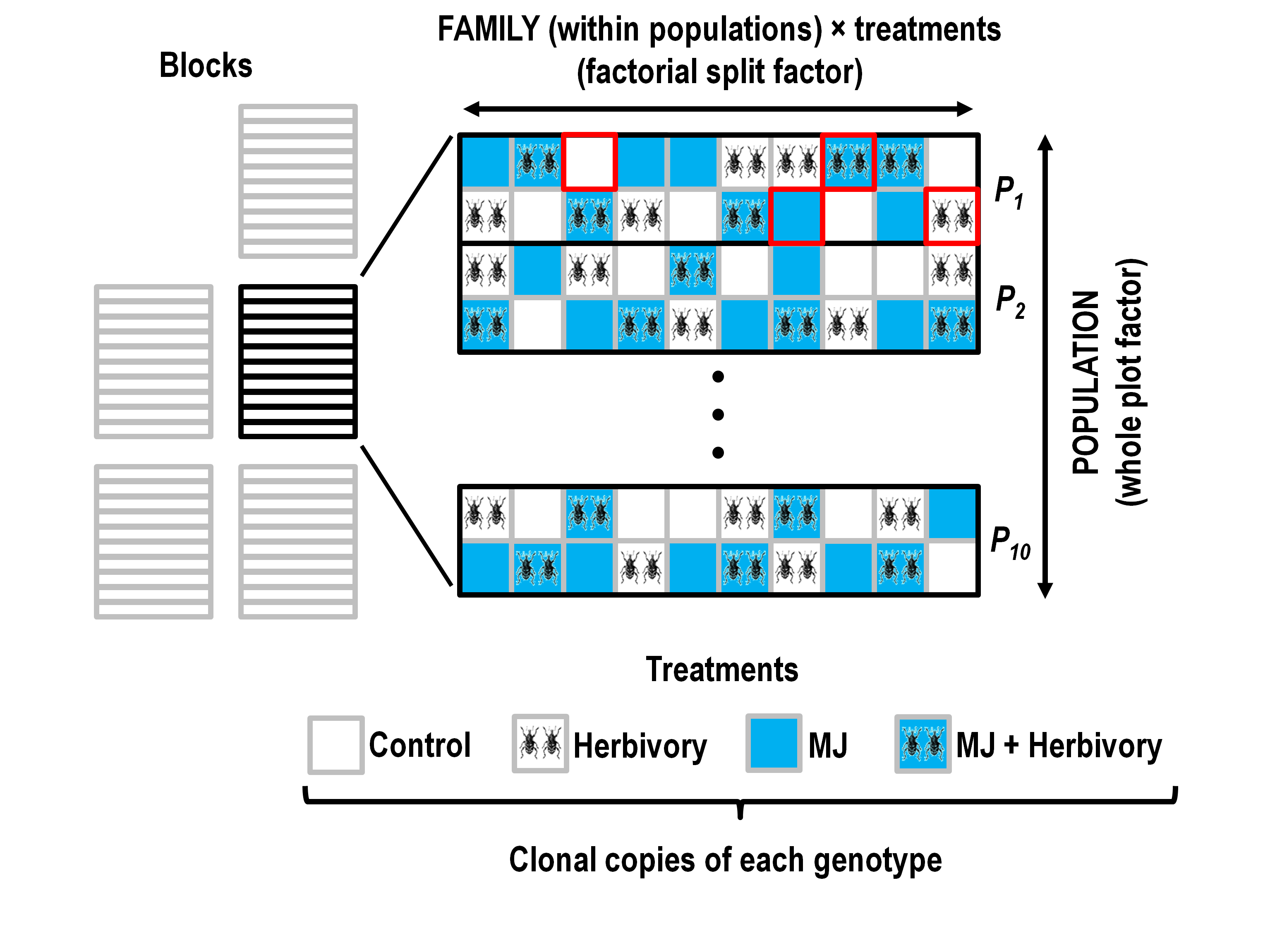


**Figure SM1.** Graphical scheme of the experimental design, showing the design within one of the five blocks. Plants were distributed in a factorial split-plot design. Population (*P_n,_ n* = 10 populations) was the whole plot factor, and the factorial combination of family (3-5 families per population) and induction treatments (MJ and herbivory) was the split factor. Squares highlighted in red depict the four clonal replicates of the same genotype from a family within a population, each one randomly allotted to the combination of the induction treatments.

**References**

Adams, R.P. (2007). *Identification of Essential Oil Components by Gas Chromatography/Mass Spectrometry.* Carol Stream, USA: Allured Publishing Corporation.

Benjamini, Y., and Hochberg, Y. (1995). Controlling the False Discovery Rate: a practical and powerful approach to multiple testing. *Journal of the Royal Statistical Society: Series B (Statistical Methodology)* 57(1)**,** 289-300.

Chancerel, E., Lepoittevin, C., Provost, G.L., Lin, Y.C., Jaramillo-Correa, J.P., Eckert, A.J., et al. (2011). Development and implementation of a highly-multiplexed SNP array for genetic mapping in maritime pine and comparative mapping with loblolly pine. *BMC Genomics* 12(368). doi: 10.1186/1471-2164-12-368.

Earl, D.A., and vonHoldt, B.M. (2011). STRUCTURE HARVESTER: a website and program for visualizing STRUCTURE output and implementing the Evanno method. *Conservation Genetics Resources* 4(2)**,** 359-361. doi: 10.1007/s12686-011-9548-7.

Hardy, O., and Vekemans, X. (2002). SPAGeDi: a versatile computer program to analyse spatial genetic structure at the individual or population levels. *Molecular Ecology Notes* 2**,** 618-620. doi: 10.1046/j.1471-8278.

Jakobsson, M., and Rosenberg, N.A. (2007). CLUMPP: a cluster matching and permutation program for dealing with label switching and multimodality in analysis of population structure. *Bioinformatics* 23(14)**,** 1801-1806. doi: 10.1093/bioinformatics/btm233.

Jaramillo-Correa, J.P., Rodriguez-Quilon, I., Grivet, D., Lepoittevin, C., Sebastiani, F., Heuertz, M., et al. (2015). Molecular proxies for climate maladaptation in a long-lived tree (*Pinus pinaster* Aiton, Pinaceae). *Genetics* 199(3)**,** 793-807. doi: 10.1534/genetics.114.173252.

Loiselle, B., Sork, V., Nason, J., and Graham, C. (1995). Spatial genetic structure of a tropical understory shrub, *Psychotria officinalis* (Rubiaceae). *American Journal of Botany* 82(11)**,** 1420-1425. doi: 10.1002/j.1537-2197.1995.tb12679.x.

Plomion, C., Bartholome, J., Lesur, I., Boury, C., Rodriguez-Quilon, I., Lagraulet, H., et al. (2016). High-density SNP assay development for genetic analysis in maritime pine (*Pinus pinaster*). *Mol Ecol Resour* 16(2)**,** 574-587. doi: 10.1111/1755-0998.12464.

Pritchard, J., Stephens, M., and Donnelly, P. (2000). Inference of population structure using multilocus genotype data. *Genetics* 155**,** 945-959.

Pritchard, J., and Wen, W. (2003). "Documentation for STRUCTURE Software: Version 2." Chicago: University of Chicago press.).

Raffa, K.F., Mason, C.J., Bonello, P., Cook, S., Erbilgin, N., Keefover-Ring, K., et al. (2017). Defence syndromes in lodgepole - whitebark pine ecosystems relate to degree of historical exposure to mountain pine beetles. *Plant Cell Environ* 40(9)**,** 1791-1806. doi: 10.1111/pce.12985.

Sampedro, L., Moreira, X., Llusia, J., Penuelas, J., and Zas, R. (2010). Genetics, phosphorus availability, and herbivore-derived induction as sources of phenotypic variation of leaf volatile terpenes in a pine species. *Journal of Experimental Botany* 61(15)**,** 4437-4447. doi: 10.1093/jxb/erq246.

Villari, C., Battisti, A., Chakraborty, S., Michelozzi, M., Bonello, P., and Faccoli, M. (2012). Nutritional and pathogenic fungi associated with the pine engraver beetle trigger comparable defenses in Scots pine. *Tree Physiol* 32(7)**,** 867-879. doi: 10.1093/treephys/tps056.

Yu, J., Pressoir, G., Briggs, W.H., Vroh Bi, I., Yamasaki, M., Doebley, J.F., et al. (2006). A unified mixed-model method for association mapping that accounts for multiple levels of relatedness. *Nat Genet* 38(2)**,** 203-208. doi: 10.1038/ng1702.
